# Rheumatoid Arthritis and Autoimmune Hemolytic Anemia as First Manifestation of Rhupus

**DOI:** 10.1155/2020/8870643

**Published:** 2020-12-09

**Authors:** Alejandra Espinosa-Orantes, Martha Adriana Hernandez-Vera, Jose Daniel Juarez-Villa, Gutiérrez-Espinoza Anahí Guadalupe, Guillermo Flores

**Affiliations:** Department of Internal Medicine, Division of Medicine, Hospital de Especialidades Del Centro Médico Nacional “Siglo XXI”, Instituto Mexicano Del Seguro Social, México 06720, Mexico

## Abstract

“Rhupus” syndrome is a rare condition that describes the coexistence of systemic lupus erythematosus (SLE) and rheumatoid arthritis (RA), which prevalence among patients with SLE varies from 0.01% to 9.7%. There are few reported cases of the association between autoimmune hemolytic anemia and rheumatoid arthritis with systemic lupus erythematosus (rhupus). We report a rare case of rhupus in a 29-year-old woman, associated with autoimmune hemolytic anemia.

## 1. Introduction

The term “rhupus” describes a syndrome that comprises the coexistence of systemic lupus erythematosus (SLE) and rheumatoid arthritis (RA). Lupus arthropathy is a nonerosive process and is relatively a common manifestation of SLE; however, approximately 1% of patients with SLE will develop erosive arthropathy, which is difficult to differentiate from AR arthropathy. Autoimmune hemolytic anemia (AHA) presents in approximately 1/10 patients with SLE, but is a very uncommon process in RA. The presence of rhupus and AHA is an extremely uncommon condition, with only a few cases previously reported [1–3].

## 2. Case Presentation

A 29-year-old woman, with no previous medical history, started 2 years before her presentation to our center with pain and 30–60 minutes morning stiffness in both hands, symmetric polyarticular pain, and swelling at both glenohumeral joints, all metacarpophalangeal, all proximal interphalangeal joints, both wrist and hip joints with pain improvement after movement. The signs and symptoms remitted partially with ketorolac, and she had also improvement while pregnancy 1 year ago. She denied other symptoms including fever, dry mouth or dry eye, muscular weakness, hair loss, oral ulcers, or dermal lesions.

Two weeks before admission, she presented dyspnea (mMRC 3). At arrival, she had a heart rate of 105 beats per minute, and other vital signs were within normal range. On physical examination, there was pain and palpable synovial thickening at both glenohumeral, all metacarpophalangeal, all proximal interphalangeal joints and both wrist joints. No other abnormal findings were found.

**Figure 1:**
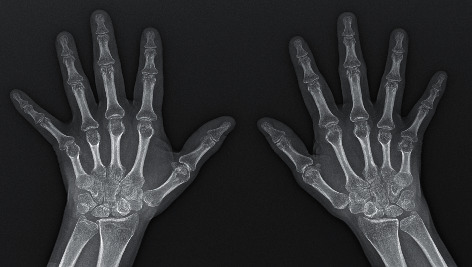
Plain radiographs of both hands.

Biochemical data showed severe anemia with elevated reticulocyte count and slightly elevated DHL, total bilirubin, and indirect bilirubin. Immunological tests were positive antinuclear antibody 1 : 2520 in an AC-1 pattern, anti-dsDNA antibody titer of 479.23 UI/ml, and anticitrullinated peptide antibody titer of 188.50 U/ml, with negative anti-Smith and antiphospholipid antibodies; other laboratory results at admission are shown in Table 1. Peripheral blood smear showed macrocytosis and punctate basophilia. Urinary sediment was not active, and no proteinuria was found. After suspected autoimmune hemolytic anemia, specific antibodies were requested, which were positive for IgG Ab directed to C3b and C3d. The plain radiograph of both hands showed juxta-articular osteopenia and joint space narrowing without bone erosions, and these findings were consistent with the articular ultrasonography of both hands, as nonerosive inflammatory arthropathy (Figure 1).

Dexamethasone was started at a dose of 40 mg for autoimmune hemolytic anemia. After two days, the patient reported significant improvement, and hemoglobin levels remained stable. She was discharged after a blood transfusion.

Diagnosis of ruphus was based on new SLE classification criteria by EULAR/ACR 2019 and on RA classification criteria revised by the EULAR/ACR. After four weeks of oral prednisone, chloroquine, and azathioprine, all symptoms and laboratory findings improved (Table 1). Initial SDAI (Simple Disease Activity Index) was 70.3, and one month after treatment, it was 0. The initial Systemic Lupus Erythematous Disease Activity Index (SLEDAI) was 8 points, and, also, after one month of treatment, it was 0 points.

## 3. Discussion

The cumulative number of globally reported rhupus cases is about 140 [1]. Among patients with SLE, rhupus syndrome has a variable prevalence from 0.01% a 9.7%; this variation is likely due to different classification and selection criteria [1–5].

The syndrome can present initially as either of both diseases, SLE or RA. The exact median onset age is not established, and the interval between RA diagnosis and development of SLE varies from 4 to 11 years [2–4]. However, Tani et al. reported a case series in which SLE features preceded arthritis onset in 50% of the patients [4].

In a cohort of Mexican population, the reported median onset age was 45.5 ± 10.7 years, and RA was the initial diagnosis in 68% of patients; it preceded rhupus for up to four years. Both were simultaneously diagnosed in the rest 32%, and no single patient was first diagnosed with SLE. In this cohort, hematologic manifestations were reported in 78%. However, AHA was seen only in one patient who initially responded to prednisone, but later, in the course of the disease, the patient developed alveolar hemorrhage and died [5]. In another study, among 56 patients with rhupus, the prevalence of AHA was just 5%, versus 21% in SLE patients [6]. Other series report no cases of rhupus and AHA [7].

Patients with rhupus tend to show a lower incidence of malar rash, renal disease, and neurological disorders. This rare condition has a lesser visceral organ involvement as compared with SLE patients without RA [5–8].

The incidence of autoimmune hemolytic anemia in RA is uncertain, but it has been described in 2.1–2.5% with 8 cases reported [9, 10].

## 4. Conclusions

Rhupus syndrome is an unusual overlap between RA and SLE. Characteristically, this syndrome shows more RA-associated manifestations and less SLE-associated damage. Autoimmune hemolytic anemia is not a common finding on RA patients, neither on rhupus cases.

We report an overlap syndrome case with an atypical initial presentation due to the presence of autoimmune hemolytic anemia, no erosive arthritis, and elevated titles of anti-CCP antibody.

## Figures and Tables

**Table 1:** Laboratory values.

Variable (normal range)	On admission	After four weeks
Creatinine (0.57–1.11 mg/dL)	0.70 mg/dL	0.66 mg/dL
Uric acid (mg/dL)	6.4 mg/dL	—
LDH (120–246 U/L)	272 U/L	189 U/L
Total bilirubin (0.00–0.90 mg/dL)	1.3 mg/dL	0.5 mg/dL
Indirect bilirubin (0.00–0.80 mg/dL)	0.9 mg/dL	0.4 mg/dL
Serum potassium (3.50–5.10 mEq/L)	3.59 mEq/L	3.73 mEq/L
Serum sodium (136–145 mEq/L)	144 mEq/L	140 mEq/L
Serum chloride (98–107 mEq/L)	107 mEq/L	107 mEq/L
Hemoglobin (13–18 g/dL)	5.6 g/dL	12.8 g/dL
Hematocrit (42–53.6%)	18.1%	39.2%
Platelets (150–450 × 10^3^/*μ*L)	337 × 10^3^/*μ*L	318 × 10^3^/*μ*L
MCV (80–97 fL)	99.8 fL	99.5 fL
MCH (27–31 pg)	31.1 pg	32.5 pg
Reticulocyte count (0.8–2.3%)	22.2%	2.8%
Erythrocyte sedimentation rate (mm/h)	10 mm/h	33 mm/h
C3 (90–170 mg/dL)	71 mg/dL	95.60 mg/dL
C4 (12–36 mg/dL)	11.70 mg/dL	23.20 mg/dL
C-reactive protein (0-00–1.00 mg/dL)	1.26 mg/dL	0.31 mg/dL
Rheumatoid factor (0.00–14.00 IU/mL)	61.8 IU/mL	—
Fibrinogen (mg/dL)	300 mg/dL	—
Iron (33–193 *μ*g/dL)	153 *μ*g/dL	—
Ferritin (4–104.2 ng/mL)	258 ng/mL	—
Direct Coombs test	Positive	—
Serologic findings (direct antiglobulin test or antibody screen results)	IgG+, C3b, C3d	—
Antinuclear antibody	1 : 2520, homogeneous pattern	—
Anti-dsDNA antibody (0–200 IU/mL)	479.23 IU/mL	205.63
Anticitrullinated peptide antibody (U/mL)	188.50 IU/mL	
Anti-Smith antibody	Negative	
Antiphospholipid antibody	Negative	
Anti-beta 2 glycoprotein 1	—	Negative (IgG, IgM)
Lupus anticoagulant antibody	—	Negative

LDH, lactate dehydrogenase; MCV, mean corpuscular volume; MCH, mean corpuscular hemoglobin; C3, complement 3; C4, complement 4; Anti-dsDNA antibodies, anti-double-stranded DNA antibodies.

## Data Availability

The data used to support the findings of this study are available from the corresponding author upon request.

## References

[1] Panush R. S., Edwards N. L., Longley S., Webster E. (1988). “Rhupus” syndrome. *Archives of Internal Medicine*.

[2] AlFadhli S., Nizam R. (2014). Rhupus: a crosswalk between lupus and rheumatoid arthritis. *OA Arthritis*.

[3] Liu T., Li G., Mu R., Ye H., Li W., Li Z. (2014). Clinical and laboratory profiles of rhupus syndrome in a Chinese population: a single-centre study of 51 patients. *Lupus*.

[4] Tani C., D’Aniello D., Sedie A. D. (2013). Rhupus syndrome: assessment of its prevalence and its clinical and instrumental characteristics in a prospective cohort of 103 SLE patients. *Autoimmunity Reviews*.

[5] Simón J. A., Granados J., Cabiedes J., Morales J. R., Varela J. A. (2002). Clinical and immunogenetic characterization of mexican patients with “rhupus”. *Lupus*.

[6] Li J., Wu H., Huang X. (2014). Clinical analysis of 56 patients with rhupus syndrome. *Medicine*.

[7] Brand C. A., Rowley M. J., Tait B. D., Muirden K. D., Whittingham S. F. (1992). Coexistent rheumatoid arthritis and systemic lupus erythematosus: clinical, serological, and phenotypic features. *Annals of the Rheumatic Diseases*.

[8] Fikry A., Faridin P. (2017). P214 rhupus syndrome: a case report and literature review. *Lupus Science & Medicine*.

[9] Estrada C. A., Lyons S., Terebelo H. (1990 May). Autoimmune hemolytic anemia and rheumatoid arthritis. *Southern Medical Journal*.

[10] Sokol R. J., Hewitt S., Stamps B. K. (1981). Autoimmune haemolysis: an 18-year study of 865 cases referred to a regional transfusion centre. *British Medical Journal*.

